# CTHRC1+ fibroblasts are stimulated by macrophage‐secreted SPP1 to induce excessive collagen deposition in keloids

**DOI:** 10.1002/ctm2.1115

**Published:** 2022-12-08

**Authors:** Jing Liu, Yan Huang, Yiyi Gong, Qingmei Liu, Jinran Lin, Jianlan Liu, Mengguo Liu, Jia Huang, Weilin Pu, Yanyun Ma, Yuting Zhang, Haiyang Li, Xiangguang Shi, Yue Zhang, Jian Wang, Yifei Zhu, Qin Wang, Kelu Wei, Jiayi Wang, Yu'ou Sha, Jiucun Wang, Wenyu Wu

**Affiliations:** ^1^ Department of Dermatology, Huashan Hospital Fudan University Shanghai China; ^2^ Human Phenome Institute, Zhangjiang Fudan International Innovation Center Fudan University Shanghai China; ^3^ State Key Laboratory of Genetic Engineering, School of Life Science Fudan University Shanghai China; ^4^ Department of Dermatology Jing'an District Central Hospital Shanghai China; ^5^ Academy for Engineering and Technology Fudan University Shanghai China; ^6^ Research Unit of Dissecting the Population Genetics and Developing New Technologies for Treatment and Prevention of Skin Phenotypes and Dermatological Diseases Chinese Academy of Medical Sciences Shanghai China

Dear Editor,

Fibrosis is characterized by fibroblast dysfunction and excessive deposition of cell‐matrix that affect the normal functioning of the original tissue or organ.^1^ The pathogenesis of keloids is complex and remains elusive. Multiple studies have suggested that skin color and tension,^2^ immunity,^3^ hormones, inflammatory stimulation,^4^ genes^5^ and other factors play critical roles in the onset and development of keloids. In addition to fibroblasts, immune cells that include macrophages,^6^ mast cells^7^ and lymphocytes^8^ are also important to keloid pathogenesis via cytokine secretion and the triggering of abnormal signaling pathways.^9^, ^10^ Therefore, performing single‐cell RNA‐sEquation (scRNA‐seq) that can identify heterogenous cell populations and cellular developmental pathways is thus valuable, as it will provide insight into the key pathogenic cell‐types and the molecular patterns.

In this study, we performed scRNA‐seq on six pairs of keloid samples to elucidate the pathogenesis of skin fibrosis. Detailed methods and single‐cell sequencing analysis are described in the Supplemental materials.

We obtained lesion and non‐lesion skin biopsies from six keloid patients (Figure 1A, Figure S1A,B, Table S1) and performed hematoxylin and eosin (H&E) and Masson staining (Figure 1B). Then we applied single‐cell sequencing (scRNA‐seq) to 12 skin samples that included both lesion and non‐lesion areas (Figure 1C). After stringent quality‐control procedures, 60,732 high‐quality cells were obtained for further analysis. We first visualized all subgroups by uniform manifold approximation and projection (UMAP) and then determined the different cell types that included fibroblasts, keratinocytes, endothelial cells, T cells, pericytes, myeloid cells, mast cells, B cells, adipocytes, melanocytes and salivary gland cells (Figure 1C,D). All cell types were identified in each sample, and we observed minimal batch effects in our study (Figure S1C‐–F). Top‐marked signatures for each cell type showed unique transcriptomic patterns in accordance with their physical functions (Figure 1E,F). We noted that extracellular matrix protein (ECM) genes were significantly enriched in fibroblasts, endothelial cells and pericytes—indicating an important role for these cell types in keloids (Figure S1G).

**FIGURE 1:**
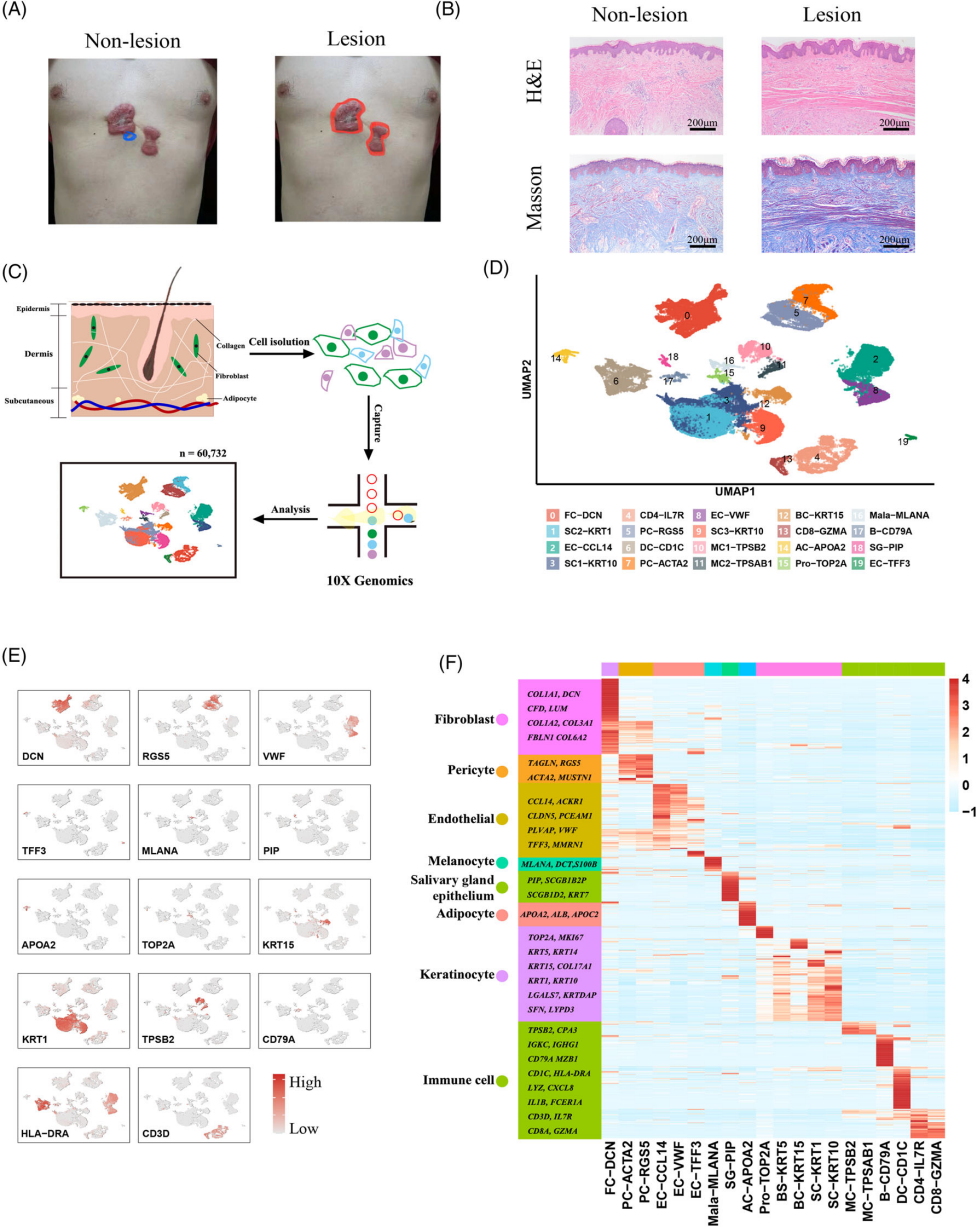
Analysis of cell types and molecular patterns of skin tissues at single‐cell resolution. (A) Schematic diagram detailing the sampling regimen employed in this study. Tissues (dermis and epidermis) taken from the blue‐encircled area were regarded as non‐lesion samples (left), while those taken from the red‐encircled area were regarded as lesion samples (right). (B) H&E and Masson staining of lesion regions and non‐lesion regions. (C) Flowchart of single‐cell sequencing. Skin tissue was separated into dermis and epidermis by dispase, followed by cellular dissolution, achieving a single‐cell suspension. Single‐cell sequencing was performed by 10X Genomics, and a total of 60 732 high‐quality cells were attained. (D) Uniform manifold approximation and projection (UMAP) plot of total cells. Cells were divided into 20 subgroups with identified molecules. FC, fibroblast cells; SC, spinous cells; EC, endothelial cells, CD4, CD4+ T cells; PC, pericytes; DC, dendritic cells; MC, mast cells; BC, basal cells; CD8, CD8+ T cells; AC, adipose cells; Pro, proliferative cells; Mala, melanocytes; B, B cells; and SG, sweat glands. (E) Feature plot of each cell type. Dark‐red colors signify elevated expression, while gray colors denote attenuated expression. (F) Heatmap of top expressed genes in eight primary cell types. Light‐blue colors signify low gene expression, while red colors denote elevated gene expression.

Fibroblasts are considered the primary source of ECM in keloids. We applied UMAP analysis to all fibroblasts (Figure 2A). Based on their expression profiles, we classified fibroblasts into five subgroups: *F01‐CCL19, F02‐CTHRC1, F03‐APCDDA, F04‐FGFBP2* and *F05‐TNN* (Figure 2B, Figure S2A). Intriguingly, ECM gene modules were significantly activated in F02 relative to other genes, as exemplified by *CTHRC1* (Figure 2C); and dual‐fluorescence staining also confirmed that CTHRC1 was co‐expressed with COL1A1/α‐SMA (Figure 2D, Figure S2B). To further substantiate that CTHRC1+ fibroblasts comprised the main source of collagen production, we separated CTHRC1+ cells from the fibroblasts (CD90+) of another three skin samples and found that they exhibited higher expression of *COL1A1* and *COL1A2* compared to the other genes (Figure 2E, Figure S2C). Furthermore, when we performed trajectory analysis to elucidate the dynamic relationships among fibroblast subgroups, our data suggested that F01 differentiated into F02 and F03 (Figure 2F), while F04 and F05 extended minimally into the trajectory (Figure S2D). The expression of ECM‐related genes was gradually augmented in the transition from F01 to F02 fibroblasts, further confirming the role of F02 fibroblasts in collagen production and keloid pathogenesis (Figure S2E).

**FIGURE 2:**
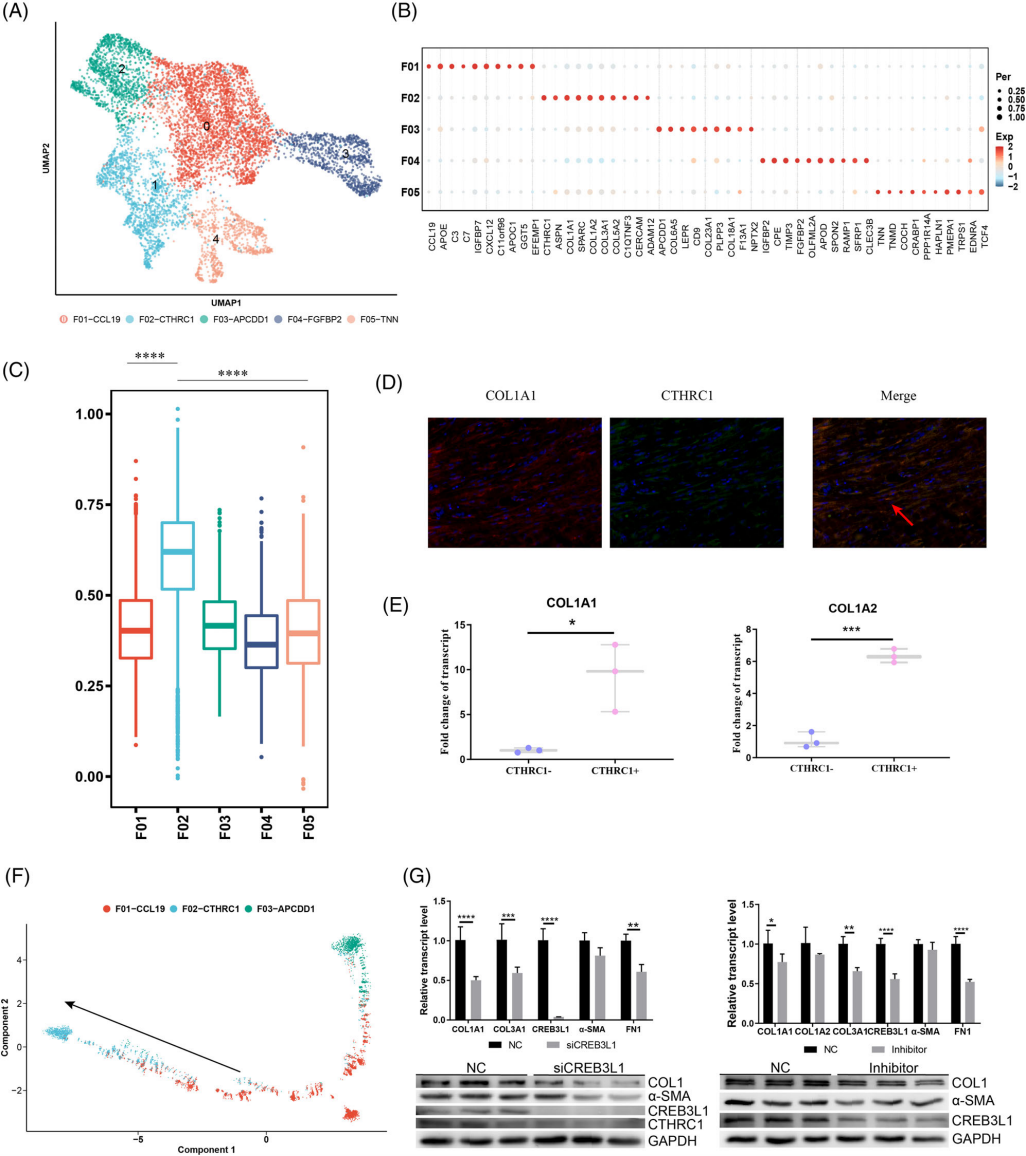
Characteristics of fibroblasts at high resolution. (A) Uniform manifold approximation and projection (UMAP) plot of fibroblasts at high resolution. Fibroblasts were divided into the five subgroups F01–F05, with each one a different color. (B) Top molecular patterns in the five subgroups. The red color designates relatively high expression, while the blue color denotes relatively low expression. The size of the circle illustrates the percentage of cells expressing the gene. (C) Gene expression of ECM model in the five subgroups based on gene ontology (GO). (D) Co‐localization staining of CTHRC1 and COL1A1 in skin tissues. Green colors designate cells expressing COL1A1, and red colors represent cells expressing CTHRC1. The red arrow shows that COL1A1 and CTHRC1 were co‐expressed in some cells. (E) CTHRC1+ cells were isolated by flow cytometry, and we found that they exhibited a higher expression of COL1A1 and COL1A2. *p < .05, ***p < .001. (F) Trajectory analysis of F01–F03. F01 may transform to F02 and F03 with pseudo‐time. (G) Gene expression of ECM‐related genes in keloid fibroblasts in which CREB3L1 interference was compared with controls; the corresponding western blot is shown at the bottom. The two‐way analysis of variance (ANOVA) was performed in this plot. (I) Gene expression of ECM‐related genes in keloid fibroblasts in which SGC‐CBP30 interference was compared with controls; the corresponding western blot is shown at the bottom. The two‐way ANOVA was performed in this plot.

In addition, we demonstrated that F02‐CTHRC1 fibroblasts were highly enriched in the lesion samples based on the ratio of observed‐to‐expected cell numbers (Ro/e) (Figure S3A), which was further validated by IHC staining (Figure S3B). Thereafter, we performed differential gene expression (DGE) analysis and uncovered several ECM‐related genes as significantly over‐expressed in lesion samples (Figure S3C,D, Table S2). Gene set variation analysis (GSVA) enrichment analysis showed that hedgehog signaling, epithelial mesenchymal transition (EMT) signaling and transforming growth factor (TGF)‐β signaling were activated in F02 fibroblasts, but not others, from lesion samples (Figure S3E); evaluation of gene‐module expression revealed that the EMT and the TGF‐β‐signaling pathways were significantly elevated in the lesion regions (Figure S3F). Trajectory analysis showed that two key genes, *THY1* (CD90) and *CTHRC1*, were gradually activated in pseudotime from non‐lesion to lesion regions (Figure S3G,H).

To clarify the factors regulating CTHRC1+ fibroblasts, we implemented SCENIC analysis and found that multiple transcription factors (TFs) were activated in CTHRC1+ fibroblasts, including *GATA2, SOX7, JDP2, IRF4* and *CREB3L1* (Figures S4A and S5A). Through regulon specificity score (RSS) score and module analysis, we determined the M9 module that is comprised of *CREB3L1, NR1D1, JDP2, MYLK* and *CREB5* might be key to the alterations observed in CTHRC1+ fibroblasts (Figures S4B–‐G and S5A‐–D). To further illustrate the regulation of TFs, we performed both siCREB3L1 and inhibitor (SGC‐CBP300 targets CREBBP/EP300) experiments in primary fibroblasts separated from the lesion regions. We found that the down‐regulation of CTHRC1 protein levels in the siCREB3L1 group was coordinated by CREB3L1, which can significantly attenuate fibrosis (Figure 2G).

Immune cells infiltrate into lesion regions to participate in skin wound healing (Figure 3A,B, Figure S6A,B). To examine the heterogeneity of macrophages in keloids, we re‐clustered macrophages and identified three cell types: Mon‐S100A8, Mac‐C1QA and Mac‐APOC1 (Figure 3C,D, Figure S6C,D). We found that most Mac‐APOC1 (SPP1 high) cells came from lesion regions and that the Mac‐C1QA cells came from lesion regions expressing COL1A1 (Figure 3E,F), where SPP1 and APOE are secreted to cooperate with fibroblasts (Figure 3G, Figure S6E). We further stimulated fibroblasts with SPP1 in vitro and showed that it enhanced collagen‐related protein expression of fibroblasts (α‐SMA, COL1A1) in a dose‐dependent manner and that it increased the proportions of CTHRC1+ fibroblasts (i.e., CTHRC1 and CREB3L1) (Figure 3H, Figure S6F).

**FIGURE 3:**
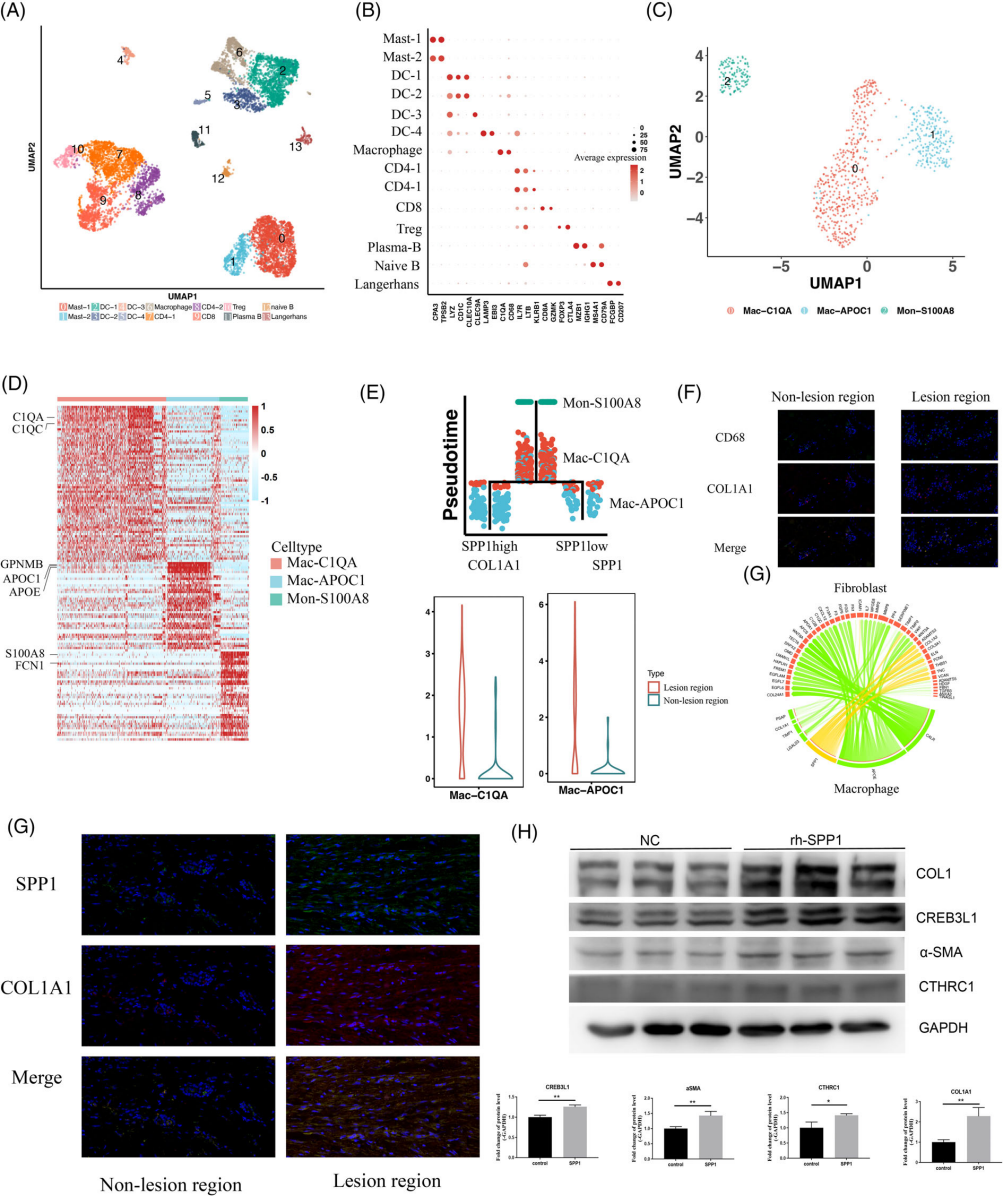
Involvement of macrophages in the pathogenesis of ECM deposition. (A) Uniform manifold approximation and projection (UMAP) plot of immune cells. Immune cells (including myeloid cells and T cells) were divided into 14 subgroups. (B) The marked molecules of each subtype are represented by dot plots. (C) UMAP plot of macrophages. Macrophages were further subdivided into three subtypes with marker molecules. (D) Heatmap of differentially expressed genes among the three macrophage subtypes. (E) Trajectory analysis of the three types of macrophages with pseudo‐time indicated that monocytes were the original cells, followed by transformation into SPP1‐low and ‐high‐expressing macrophages. Expression of COL1A1 between non‐lesion and lesion regions in Mac‐C1QA is shown at the bottom‐left, and the expression of SPP1 between non‐lesion and lesion regions in Mac‐APOC1 is shown at the bottom‐right. (F) Interaction of macrophages and fibroblasts based on molecular patterns indicated that macrophages interact with fibroblasts via SPP1. (G) Co‐localization staining of SPP1 and COL1A1 in non‐lesion and lesion regions. SPP1 was stained with the green color, while COL1A1 was stained with the red color. (H) Western blot of ECM‐related proteins with simulation by SPP1 in vitro

When we investigated the interactions among all the cell types, we ascertained that monocyte/macrophage cells, endothelial cells, pericytes and fibroblast cells interacted more robustly than the other cell types (Figure 4A–‐C). Fibroblasts interacted with endothelial cells via *ACKR1*, while multiple chemokines (*CXCL2*, *CCL3L3*) secreted by myeloid cells interacted with *DPP4* in fibroblasts (Figure 4D).

**FIGURE 4:**
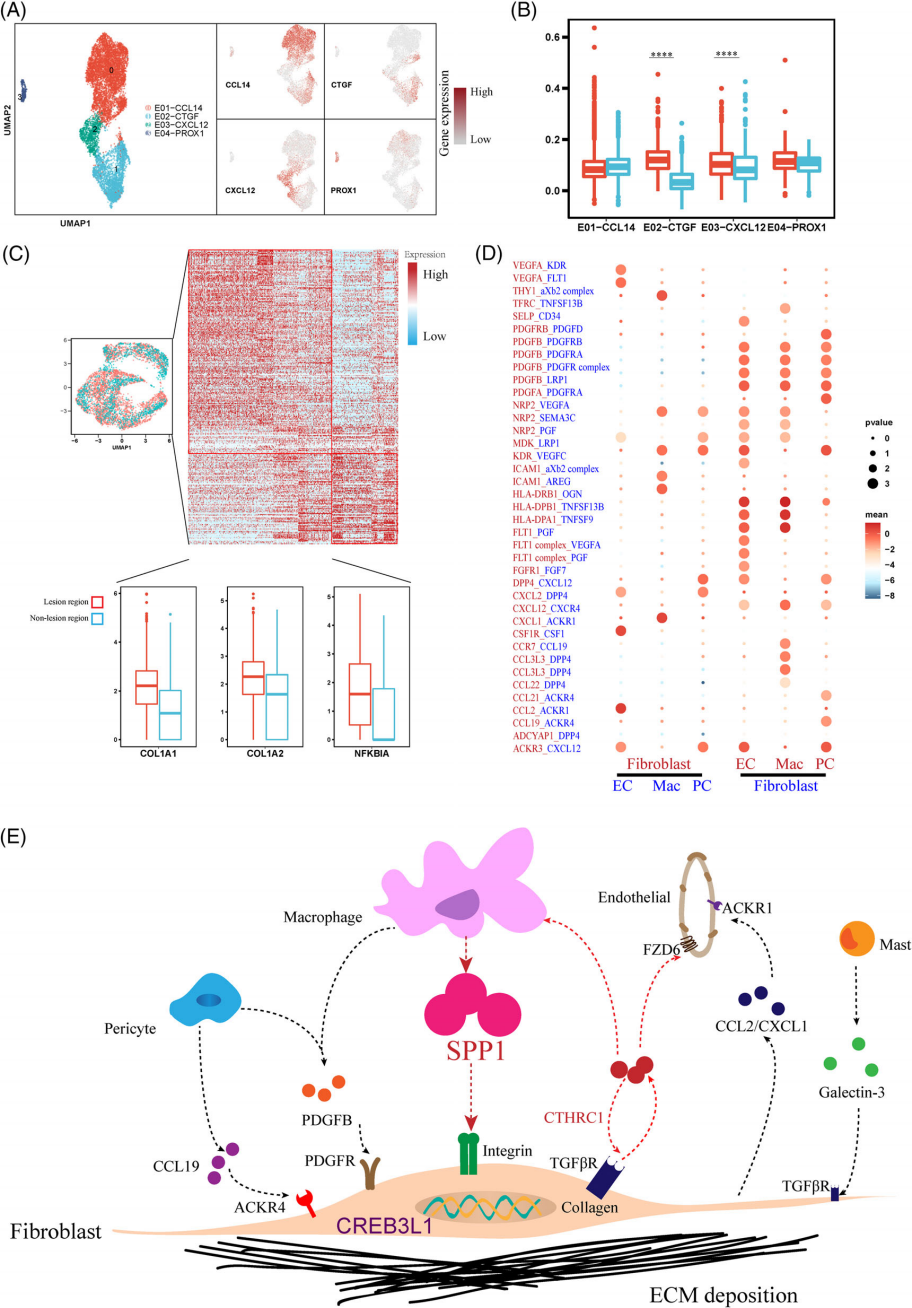
Interaction network of macrophages, endothelial cells, pericytes and fibroblasts. (A) Uniform manifold approximation and projection (UMAP) plot of endothelial cells. Feature plot of marker molecules is shown at right. (B) Expression of ECM‐pathway molecules between non‐lesion and lesion regions in four endothelial subtypes. (C) Differentially expressed genes of non‐lesion and lesion regions in pericytes. Expression of COL1A1, COL1A2 and NFKBIA is shown at the bottom. (D) Ligand‐receptor interactions between fibroblasts and the other three cell types (macrophages, endothelial cells and pericytes) using CellPhoneDB. (E) Schematic diagram illustrating the pathogenesis of skin fibrosis. The result from the database of ligand‐interaction pairs (UniProt, Ensembl, PDB, the IMEx consortium, IUPHAR)

As described above, we demonstrated a major contribution by CTHRC1+ fibroblasts in collagen deposition and that macrophages infiltrate into the lesion regions; we thus inferred that macrophages communicated with CTHRC1+ fibroblasts to further enhance collagen deposition. We then focused on the ECM pathway in CTHRC1+ fibroblasts and found that the SPP1 that was principally produced by Mac‐APOC1 increased ECM expression from CTHRC1+ fibroblasts. Collectively, our results reveal that CTHRC1+ fibroblasts occupy a vital role in excessive collagen deposition forming a positive loop with myeloid cells, endothelial cells and pericytes in this pathologic phenomenon, which carries the potential to become a targeted cell‐type in treatment strategies (Figure 4E).

## FUNDING INFORMATION

National Natural Science Foundation of China, Grant Numbers: 82173442, 82001726 and 81301643; Shanghai Engineering Research Center of Hair Medicine, Grant Number: 19DZ2250500; Shanghai Municipal Science and Technology Major Project, Grant Number: 2017SHZDZX01; Chinese Academy of Medical Sciences, Grant Number: 2019‐I2M‐5‐066; National Postdoctoral Program for Innovative Talents, Grant Number: BX20190076; Jing'an District Clinical Advantage Special Disease Construction Project, Grant Number: 2021ZB01; Shanghai Health Leading Talent, Grant Number: 2022LJ017.

## CONFLICT OF INTEREST

All authors declared no conflict of interest.

## Supporting information

Figure S1

Figure S2

Figure S3

Figure S4

Figure S5

Figure S6

Supporting Information

Table S1

Table S2

Table S3
