# Supplementary figures and images for: CTHRC1+ fibroblasts are stimulated by macrophage‐secreted SPP1 to induce excessive collagen deposition in keloids
Source: Clin Transl Med. 2022 Dec 8;12(12):e1115. doi: 10.1002/ctm2.1115 (PMC9731391; doi:10.1002/ctm2.1115)

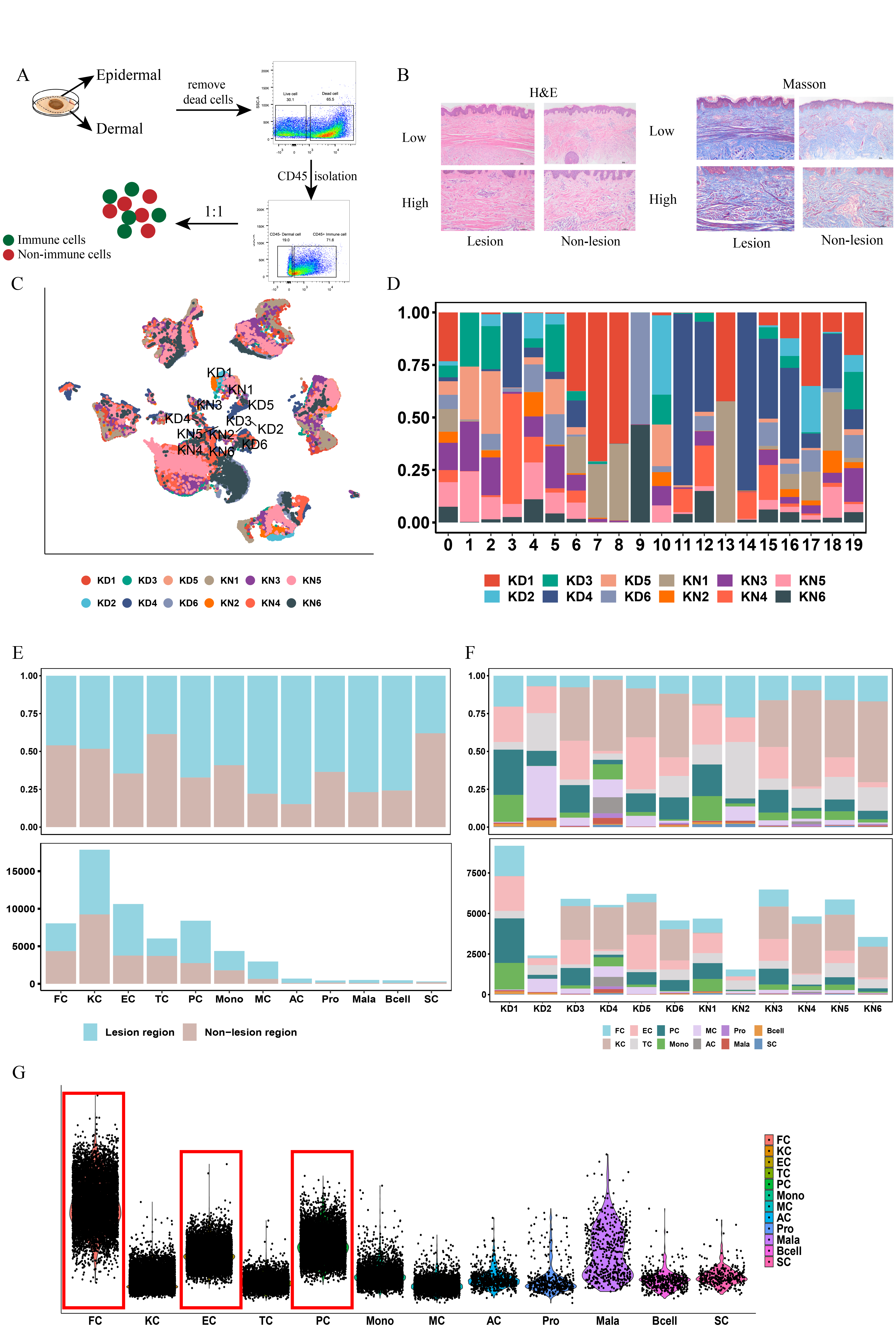

Supplement: Supplementary file 1 — Figure S1 [file CTM2-12-e1115-s010.png]

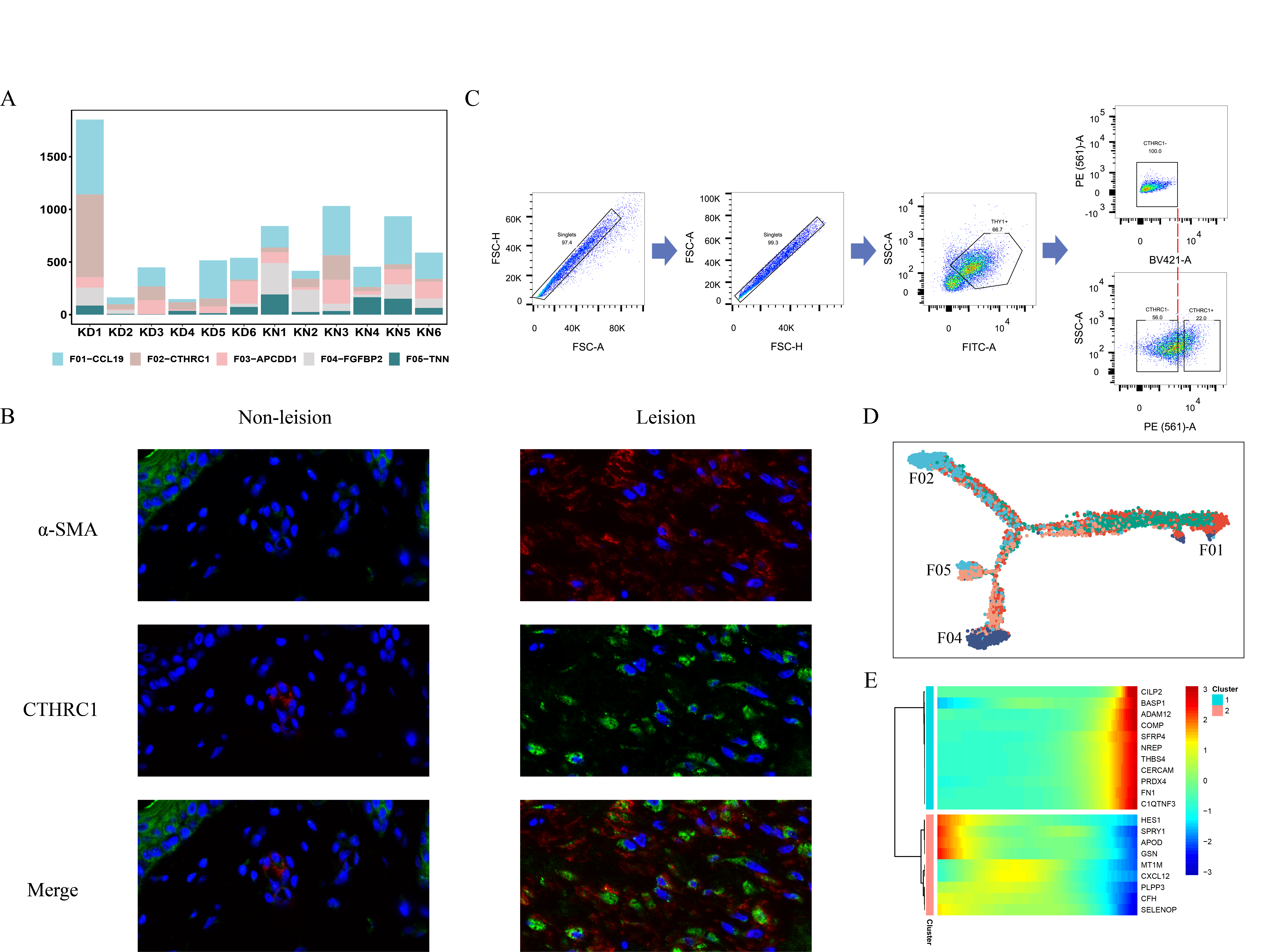

Supplement: Supplementary file 2 — Figure S2 [file CTM2-12-e1115-s005.png]

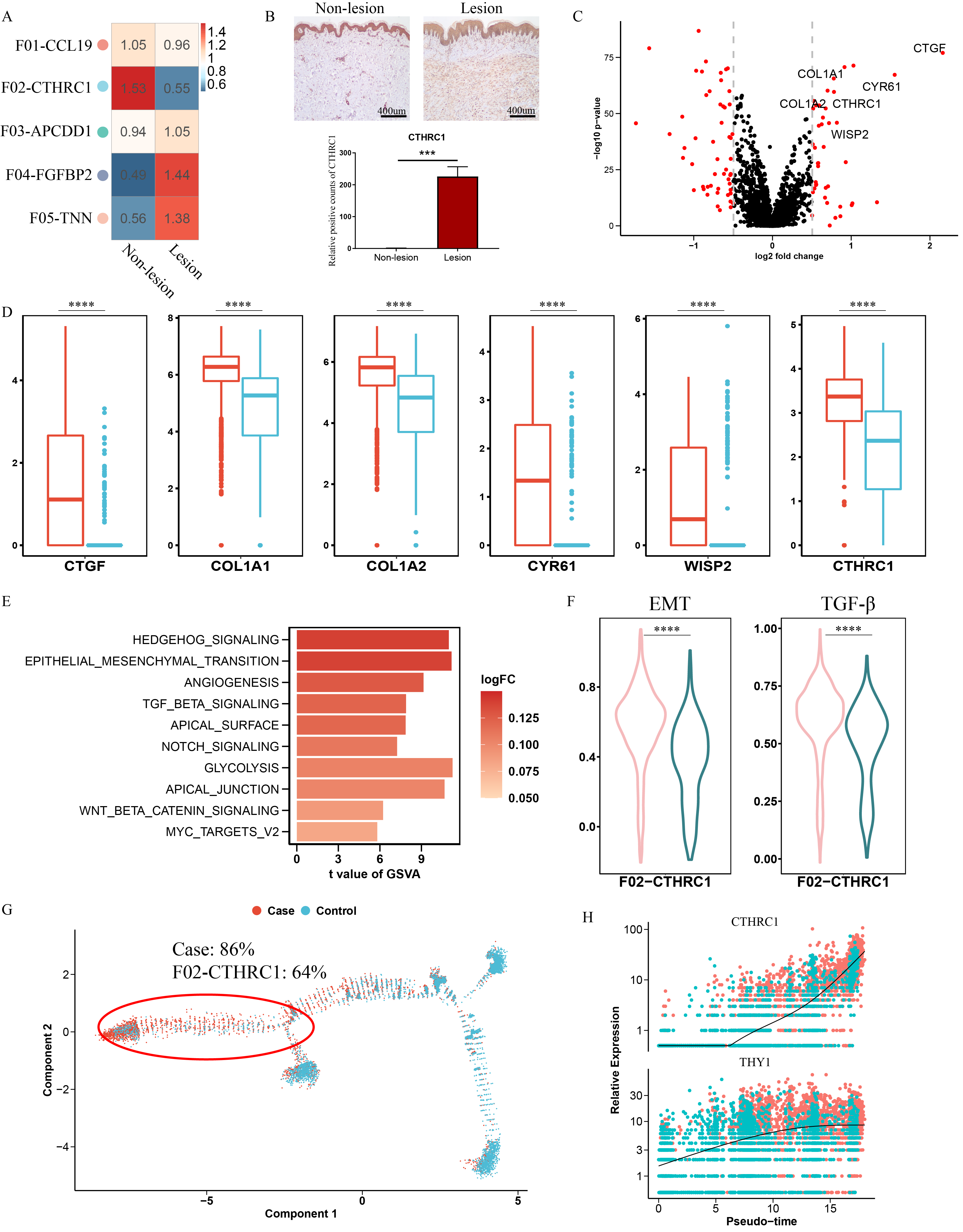

Supplement: Supplementary file 3 — Figure S3 [file CTM2-12-e1115-s003.png]

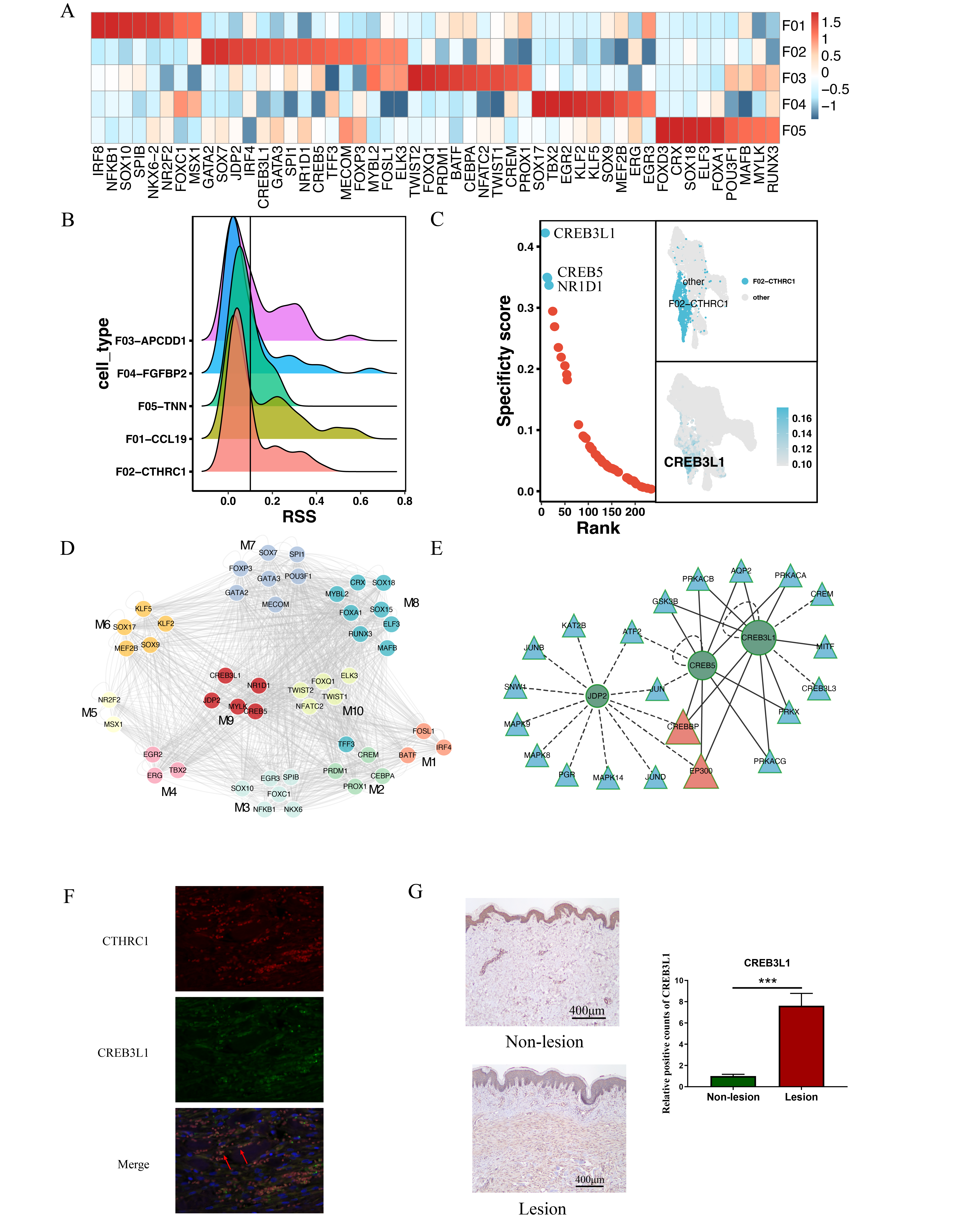

Supplement: Supplementary file 4 — Figure S4 [file CTM2-12-e1115-s002.png]

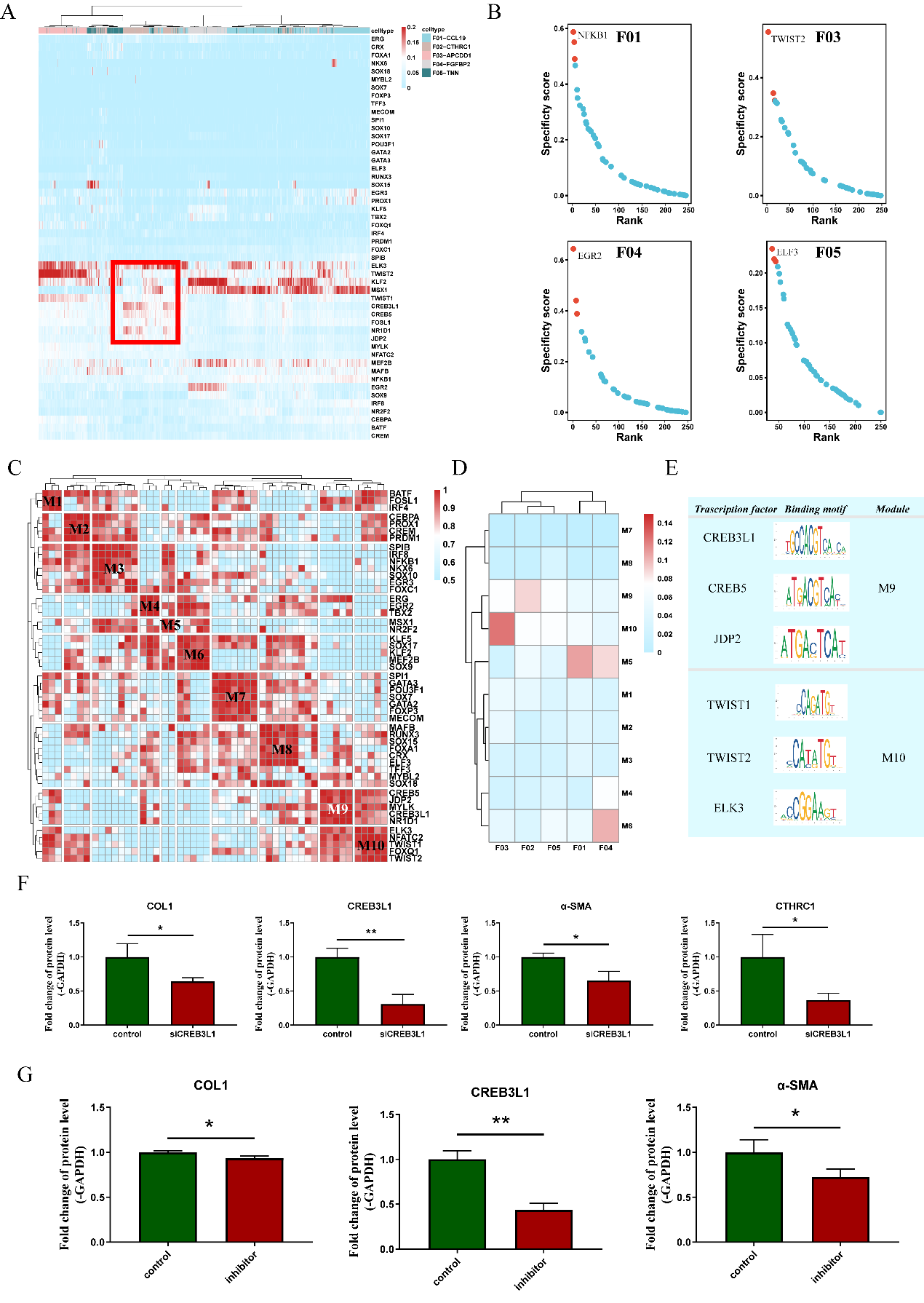

Supplement: Supplementary file 5 — Figure S5 [file CTM2-12-e1115-s008.png]

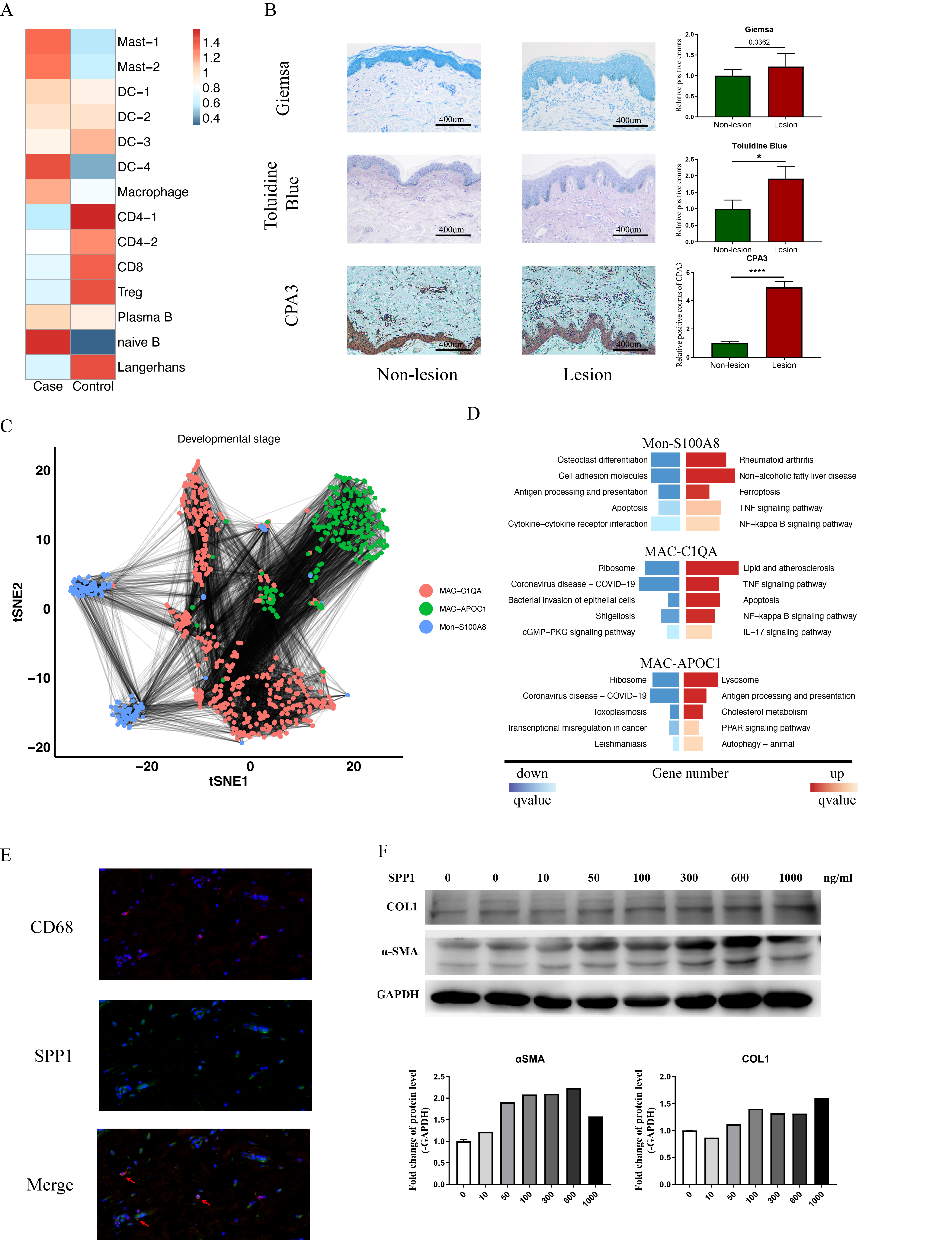

Supplement: Supplementary file 6 — Figure S6 [file CTM2-12-e1115-s007.png]
