# Supplementary material for: CTHRC1+ fibroblasts are stimulated by macrophage‐secreted SPP1 to induce excessive collagen deposition in keloids
Source: Clin Transl Med. 2022 Dec 8;12(12):e1115. doi: 10.1002/ctm2.1115 (PMC9731391; doi:10.1002/ctm2.1115)
Supplement: Supplementary file 7 — Supporting Information [file CTM2-12-e1115-s006.docx]

**METHODS**

**Human specimens**

For the present study, we enrolled six patients with keloids who underwent surgery at Huashan Hospital and collected a total of 12 samples—six from non-lesion regions and six from lesion regions. The clinical information that depicts patient demographics and disease sites is presented in Table S1. This study was approved by the Ethics Committee of Huashan Hospital, and patients provided informed consent prior to surgery. None of our patients indicated a history of drug use or other treatments before surgery, or manifested any serious diseases.

**Sample preparation**

Surgically obtained skin tissue was immediately washed three times with phosphate- buffered saline (PBS, Sigma-Aldrich) containing 2% penicillin and streptomycin (to clear impurities such as blood clots) and stored in ice-cold PBS for immediate transfer to the laboratory for cell isolation. After the skin tissue was washed twice with cold PBS, adipose tissues under the dermis were removed and the sample was transferred to 2-mL centrifuge tubes. After we incubated the samples with dispase II (Sigma) at 37°C in a water bath for 30 min (shaking the tubes vigorously every 10 min), we peeled off the epidermis and minced it into small pieces that were digested at 37°C for 15 min using 0.25% trypsin-EDTA. The remaining dermis was minced with scissors into ~1-mm^2^ pieces and digested at 37°C for 30 min in digestion solution (DMEM supplemented with 2.5 mg/mL collagenase P, 1 mg/mL DNase, and 1 mg/mL dispase II). The single-cell suspension was sequentially filtered through 70-μm and 40-μm strainers (BD Falcon) and centrifuged at 300 × g for 6 min at 4°C. Cells were resuspended in 5 mL of cold PBS, washed twice as mentioned above, and then resuspended in 100 μL of cold PBS with 2% FBS for flow cytometric analysis.

**Flow cytometry**

The cell suspension was incubated with Fixable Viability Dye eFluor 506 (BV510, ThermoFisher, USA) for 30 min at 4°C in PBS and then incubated with CD45 (PerCP-Cy5.5, BD Biosciences, USA) for 20 min at 4°C. To separate CTHRC1+ fibroblasts from keloid skin tissues, we incubated the cellular suspension with THY1 (C49406-AF488, SAB, USA) and CTHRC1 (C48364-AF555, SAB, USA) for 2 h at 37°C and conducted flow cytometry using a BD FACSAria III (BD Biosciences). Data were analyzed using FlowJo_V10_CL_Diag (Tree Star).

**Single-cell RNA sequencing using 10X Genomics**

We applied the Gel Bead Kit V3 (10x Genomics, Pleasanton, CA) for library preparation according to standardized protocols. The single-cell libraries were further sequenced on an Illumina NovaSeq 6000 Systems platform with paired-end sequencing.

**Analysis of scRNA-seq data**

1. Quality control and batch-effect correction

The gene-barcode matrices were generated with the Cell Ranger toolkit (v3.1), the sequences were aligned to the GRCh38 human reference genome, and the unique molecular identifiers (UMIs) were counted for each cell. Thereafter, ambient RNA was excluded by SoupX^1^. We executed quality controls of the scRNA-seq data and downstream analysis using the “Seurat” R package (v3.1.4) ^2^. First, cells with a high proportion of mitochondrial gene counts (>10%), UMIs < 500, or UMIs > 5000 were filtered out; and potential doublets were removed through the DoubletFinder (v2.0) package with default settings ^3^. Next, we normalized the feature expression for each cell with “LogNormalize” and log-transformed the results with the NormalizeData() function. We then corrected the batch effect with the “Harmony” package ^4^ and ultimately obtained the scaled and batch effect-corrected expression profiles of all samples for use in subsequent analyses.

1. Unsupervised clustering and dimensional reduction

The top principal components (PCs) were computed based on the gene expression profiles of 2000 highly variable genes (HVGs), and we selected the optimal number of PCs for further analysis using the PCElbowPlot() function and applied the FindNeighbors() and FindClusters() functions for cell clustering. In order to identify the optimal resolution, a “clustering tree” method was exploited ^5^, and the RunUMAP() function was used for visualization. The cell identity of each cluster was defined by the expression of well-known markers for T cells such as *CD3D*, *CD3E*, and *IL7R*.

1. Identification of signatures for each cell cluster

The differentially expressed genes (DEGs) in each sub-cluster were identified through the FindAllMarkers() function in Seurat. We then used the Wilcoxon rank-sum test to determine the significance level of each gene and applied the following criteria to determine the signatures of each cluster: a) those expressed in more than 20% of the cells within either or both of the two groups; b) |log_2_FC| > 0.5; and c) a Wilcoxon rank-sum test adjusted *P*-value < 0.01.

**Inference of cell state by trajectory analysis**

The trajectory analysis was performed with the Monocle2 ^6^ and the URD packages ^7^ to reveal cellular transitions; the ordering of genes and variance level were set as recommended by Monocle2. We then executed the DDRTree() function in Monocle2 to reduce dimensionality with default settings and applied the differentialGeneTest() function in Monocle2 to reveal the variance in DEGs with pseudotime.

We employed the URD package to reveal the transitional process of the macrophages, and all parameters were set as defaults according to the package. We set monocytes (S100A8) as root cells and built trees with the buildTree() function.

**Construction of ECM scores in the scRNA-seq dataset**

ECM deposition constitutes the crucial precipitator of skin fibrosis. To assess the potential transcriptional activity of different cells, we constructed an ECM module based on the Gene Ontology (GO) database, with the module score computed with AddModuleScore() in the Seurat package. The list of signatures involved in the ECM pathway is presented in Table S2.

**Analysis of cell-cell interactions**

To investigate the potential interactions between fibroblasts and other ECM-producing cells, we adopted CellPhoneDB (Python package, v2.1.4), which integrates a publicly available repository of curated ligand-receptor (L-R) pairs and a statistical framework ^8^. As described above, fibroblasts, endothelial cells, pericytes, and macrophages were assessed in order to identify their possible interactions. The significant cell-type-specific interactions between L-R pairs (*P* < 0.05; principally, cytokines, chemokines, and growth factors) were selected for visualization; and to further scrutinize and visualize the interaction of macrophages and fibroblasts, we employed NicheNet according to the recommended manual protocol ^9^. NicheNet (<https://github.com/saeyslab/nichenetr>) is a method that predicts ligand–target links between interacting cells by combining their expression data with prior knowledge of signaling and gene regulatory networks.

**SCENIC and downstream analyses**

We employed the Single-Cell Regulatory Network Inference and Clustering (SCENIC) analysis to reveal the gene regulatory network (GRN) with respect to different cell types and clusters ^10^; the SCENIC analysis for this study was performed according to the latest version of the pySCENIC pipeline. The gene-motif rankings (500 bp upstream or 100 bp downstream from the transcriptional start site [TSS]) were used to determine the search space around the TSS. The motif database (mc9nr) that included 24,453 motifs was then used for RcisTarget and GENIE3 algorithms to allow inference of the GRNs. Regulon analysis was then executed to further explore the fibroblast regulons ^11^, and the regulon specificity score (RSS) was computed to determine how specific each predicted regulon was for each cell type ^12^. The connection specificity index (CSI) for all regulons was subsequently calculated to reveal the connectedness between the different regulons ^13^. We used Cytoscape to visualize the correlations of the regulons in different models (CSI > 0.7 was taken as a cut-off) ^14^.

**Transcription regulatory network**

To illustrate the functions of the regulons in the M9 and M10 gene modules, we evaluated the regulon binding motifs and putative targets. Cytoscape was used to visualize the network of regulons based on the RegNetwork and JASPAR databases ^15,16^.

**Histologic analysis**

Paraformaldehyde-fixed, paraffin-embedded sections (3–5 µm) were used for hematoxylin and eosin (H&E), Masson trichrome, immunohistochemical (IHC), and immunofluorescence (IF) staining according to standard protocols. H&E or Masson’s trichrome staining was applied to detect the dermal thickness and collagen density in skin tissues. We discriminated the mast cells in skin tissues with toluidine blue and Giemsa staining. Anti-CTHRC1 (1:200, ab85739, Abcam, UK) and -CREB3L1 antibodies (1:200, 11235-2-AP, Proteintech, USA) were used for IHC; and antibodies generated against COL1A1 (1:500, ab138492, Abcam, UK), CTHRC1 (1:250, ab85739, Abcam, UK), CREB3L1 (1:150, 11235-2-AP, Proteintech, USA), CD68 (1:100, ab213363, Abcam, UK), and SPP1 (1:1000, ab8448, Abcam, UK) were used for IF staining. All images were observed and photographed under a light microscope (Olympus, Tokyo, Japan).

**Isolation and culture of primary fibroblasts from human keloid tissues**

The primary culture of keloid fibroblasts was implemented with the tissue-block adherence method, with the following detailed isolation and culture steps. First, surgically excised tissue was placed on ice and shipped to the laboratory within 1 h. After washing twice with 100 mg/mL streptomycin in serum-free medium, the epidermis was carefully scraped with ophthalmic scissors, and the remaining tissue was washed 2–3 times with serum-free medium and minced with scissors into 0.5–1 mm^3^ pieces. We washed the pieces in serum-free medium several times until the liquid was clear, with no oil droplets evident. Next, we placed the tissue block into the culture flask for 30 min and dried the block to allow it to adhere to the surface of the culture flask. DMEM high-glucose medium containing 10%–20% fetal bovine serum or calf serum was added to the culture flask, which was then placed in a 5% CO_2_ incubator at 37°C under high humidity. We did not physically contact or observe the tissue for 3–4 days so as not to affect the adherence and growth of the tissue block. After 1 week, we replaced the culture medium once or twice daily, and cells began to grow 4–7 days after the tissue block was seeded. Cell passage was initiated when fibroblasts were in several large, dense colonies, or when fibroblasts filled the bottom of the flask.

**Gene silencing of CREB3L1 and CREBBP/EP300 inhibition**

For transfection experiments, human keloid primary fibroblasts were transfected with 0.04 nM CREB3L1-siRNA mixed with 2 μL of Lipofectamine^®^ RNAiMAX transfection reagent (Invitrogen, Thermo Fisher Scientific, USA), and the transfection was evaluated 2 days later by IF staining. Small interfering RNA (siRNA) sequences against *CREB3L1* were designed and synthesized by Genomeditech (GenePharma, Shanghai, China), and we purchased the inhibitor of CREBBP/EP300 (SGC-CBP30) from Selleck (Catalog No. S7256).

**Treatment of recombinant SPP1 protein**

For recombinant protein-stimulation experiments, dermal (foreskin) fibroblasts (HFF-1) were successively incubated with 10, 50, 100, 300, 600, or 1000 ng/mL recombinant SPP1 protein (1433-OP-050, R&D Systems, USA); and we extracted RNA and protein from HFF-1 after 24-h and 48-h incubation periods with or without recombinant SPP1 protein co-incubation.

**RNA isolation and real-time PCR**

Total RNA was extracted from the keloid fibroblasts or control HFF-1 using Trizol reagent (Invitrogen, Carlsbad, CA, USA) according to the manufacturer’s instructions, and 1 µg of total RNA was reverse-transcribed to complementary DNA (cDNA) using a High-Capacity cDNA Reverse Transcription Kit (Applied Biosystems, CA, USA). We executed real-time PCR with SYBR Premix ExTaq (Takara Biotech, Tokyo, Japan) and analyzed amplification with an ABI Prism 7900 Detector System (Applied Biosystems), using the housekeeping gene *GAPDH* as an endogenous control. The real-time RT-PCR primers are listed in Table S3.

**Western blot analysis**

According to the standardized protocol, equal amounts of protein were subjected to sodium dodecyl sulfate-polyacrylamide gel electrophoresis (SDS-PAGE), and proteins were transferred onto polyvinylidene fluoride (PVDF) membranes. The PVDF membranes were blocked in Tris-buffered saline/Tween-20 (TBST, pH 7.4) containing 5% non-fat milk for 2 h. The PVDF membranes were then incubated with their respective primary antibodies for 8 h at 4°C and then with the secondary antibody for 2 h at room temperature. After washing three times with TBST, protein bands were visualized using an enhanced chemiluminescence system, and band intensity was quantified using Image-QuantTL software (General Electric Company, CT, USA).

**Statistics analysis**

The t-test analysis was performed in experiments including H&E staining, IHC, and western blot. The two-way ANOVA was performed in comparing different groups including transcription expression level.

**Discussion**

Several groups of researchers have performed transcriptomics analyses, including two single-cell RNA sequencing studies that described the molecular patterns in keloids ^17,18^. Deng *et al.* focused on the heterogeneity of fibroblasts and indicated that mesenchymal fibroblasts contributed more to collagen deposition compared to keloids in normal scar tissues. Although mesenchymal fibroblasts possess a pathogenic role in keloids, they do not produce COL1A1 directly; studies have not clarified the interactions of fibroblasts with other cell types, especially immune cells. Therefore, our study aimed to identify the pathogenic fibroblasts that overexpress COL1A1 and COL3A1 and to elucidate the governing molecular regulatory networks and cell-cell interaction models. Such data would supplement the information derived from previous studies to uncover a potential molecular target(s) for skin fibrosis.

In this study, we first identified the major cell types in the skin tissues of keloids—including fibroblasts, endothelial cells, pericytes, and immune cells—and discovered that fibroblasts contributed the majority of the ECM matrix relative to the other cell types, followed by pericytes and endothelial cells (**Fig. S1G**). We therefore analyzed fibroblasts at a higher resolution and identified five fibroblast subtypes that were consistent with previous studies ^19-21^. Our analysis revealed that CTHRC1+ fibroblasts were significantly enriched in the lesion regions of keloids and produced more ECM than CTHRC1- fibroblasts (**Figs. 2-3**). We also determined that CTHRC1+ fibroblasts were regulated by a module of TFs (M9) that included CREB5, JDP2, MYLK, CREB3L1, and NR1D1 (**Fig. S3C**). Investigators previously suggested that mesenchymal fibroblasts exhibited molecular programs similar to those of F03-APCDD1, were significantly enriched in keloids, and were important to collagen deposition ^18^. We then integrated the authors’ dataset with ours and established that F03 cells expressed higher levels of *COL11A1* and *COL4A1* that were linked with the EndoMT. Deng *et al.* also indicated that supernatants of the mesenchymal transition could promote the collagen expression of other fibroblasts. Our results intriguingly revealed that M9 was highly linked to M10 (ELK3, NFATC2, TWIST1, FOXQ1, and TWIST2) and that both were regulons in F03-APCDD1. We suggested herein that mesenchymal fibroblasts were much more likely to cooperate with CTHRC1+ fibroblasts, which were then responsible for COL1A1 and COL3A1 production. We posit that further investigation of the relationship between these two fibroblast subtypes is warranted in the future.

**Legend**

**Fig. 1. Analysis of cell types and molecular patterns of skin tissues at single-cell resolution.** **A** Schematic diagram detailing the sampling regimen employed in this study. Tissues (dermis and epidermis) taken from the blue-encircled area were regarded as non-lesion samples (left), while those taken from the red-encircled area were regarded as lesion samples (right). **B** H&E and Masson staining of lesion regions and non-lesion regions. **C** Flowchart of single-cell sequencing. Skin tissue was separated into dermis and epidermis by dispase, followed by cellular dissolution, achieving a single-cell suspension. Single-cell sequencing was performed by 10X Genomics, and a total of 60,732 high-quality cells were attained. **D** UMAP plot of total cells. Cells were divided into 20 subgroups with identified molecules. FC, fibroblast cells; SC, spinous cells; EC, endothelial cells, CD4, CD4+ T cells; PC, pericytes; DC, dendritic cells; MC, mast cells; BC, basal cells; CD8, CD8+ T cells; AC, adipose cells; Pro, proliferative cells; Mala, melanocytes; B, B cells; and SG, sweat glands. **E** Feature plot of each cell type. Dark-red colors signify elevated expression, while gray colors denote attenuated expression. **F**: Heatmap of top expressed genes in eight primary cell types. Light-blue colors signify low gene expression, while red colors denote elevated gene expression.

**Fig. 2. Characteristics of fibroblasts at high resolution. A** UMAP plot of fibroblasts at high resolution. Fibroblasts were divided into the five subgroups F01–F05, with each one a different color. **B** Top molecular patterns in the five subgroups. The red color designates relatively high expression, while the blue color denotes relatively low expression. The size of the circle illustrates the percentage of cells expressing the gene. **C** Gene expression of ECM model in the five subgroups based on Gene Ontology (GO). **D** Co-localization staining of CTHRC1 and COL1A1 in skin tissues. Green colors designate cells expressing COL1A1, and red colors represent cells expressing CTHRC1. The red arrow shows that COL1A1 and CTHRC1 were co-expressed in some cells. **E** CTHRC1+ cells were isolated by flow cytometry, and we found that they exhibited a higher expression of COL1A1 and COL1A2. *p < 0.05, ***p < 0.001. **F** Trajectory analysis of F01–F03. F01 may transform to F02 and F03 with pseudo-time. **G** Gene expression of ECM-related genes in keloid fibroblasts in which CREB3L1 interference was compared with controls; the corresponding western blot is shown at the bottom. The two-way ANOVA was performed in this plot. **I** Gene expression of ECM-related genes in keloid fibroblasts in which SGC-CBP30 interference was compared with controls; the corresponding western blot is shown at the bottom. The two-way ANOVA was performed in this plot.

**Fig. 3. Involvement of macrophages in the pathogenesis of ECM deposition. A** UMAP plot of immune cells. Immune cells (including myeloid cells and T cells) were divided into 14 subgroups. **B** The marked molecules of each subtype are represented by dot plots. **C** UMAP plot of macrophages. Macrophages were further subdivided into three subtypes with marker molecules. **D** Heatmap of differentially expressed genes among the three macrophage subtypes. **E** Trajectory analysis of the three types of macrophages with pseudo-time indicated that monocytes were the original cells, followed by transformation into SPP1-low and -high-expressing macrophages. Expression of COL1A1 between non-lesion and lesion regions in Mac-C1QA is shown at the bottom-left, and the expression of SPP1 between non-lesion and lesion regions in Mac-APOC1 is shown at the bottom-right. **F** Interaction of macrophages and fibroblasts based on molecular patterns indicated that macrophages interact with fibroblasts via SPP1. **G** Co-localization staining of SPP1 and COL1A1 in non-lesion and lesion regions. SPP1 was stained with the green color, while COL1A1 was stained with the red color. **H** Western blot of ECM-related proteins with simulation by SPP1 in vitro.

**Fig. 4. Interaction network of macrophages, endothelial cells, pericytes, and fibroblasts. A** UMAP plot of endothelial cells. Feature plot of marker molecules is shown at right. **B** Expression of ECM-pathway molecules between non-lesion and lesion regions in four endothelial subtypes. **C** Differentially expressed genes of non-lesion and lesion regions in pericytes. Expression of COL1A1, COL1A2, and NFKBIA is shown at the bottom. **D** Ligand-receptor interactions between fibroblasts and the other three cell types (macrophages, endothelial cells, and pericytes) using CellPhoneDB. **E** Schematic diagram illustrating the pathogenesis of skin fibrosis. The result from the database of ligand-interaction pairs (UniProt, Ensembl, PDB, the IMEx consortium, IUPHAR)

**Fig. S1. Basic characteristics of sc-RNA sequencing data. A** The process of collecting samples and single-cell isolation. We separated dermal and epidermal cells, digested the cells with enzymes, removed dead cells, isolated CD45+ and CD45- cells, and finally mixed CD45+ and CD45- cells in the ratio 1:1. **B** H&E and Masson staining of non-lesion regions and lesion regions at low and high resolution. **C** UMAP plot of total cells; colors represent different samples. KD1–KD6 were sampled from lesion regions, and KN1–KN6 were sampled from non-lesion regions. **D** The cell percentage for each sample in different clusters. **E** The number (top) and percentage (bottom) of cells from non-lesion regions and lesion regions clustered by different cell types. FC, fibroblast cells; KC, keratinocytes; EC, endothelial cells; TC, T cells; PC, pericytes; mono, monocytes/macrophages; MC, mast cells; AC, adipose cells; Pro, proliferative cells; Mala, Melanocytes; Bcell, B cells; and SC, sweat gland cells. **F** The number (top) and percentage (bottom) of different cell types clustered by different samples. **G** Expression of ECM-pathway molecules in different cell types. Red rectangles designate the top three cell types.

**Fig. S2. Identification of CTHRC1+ fibroblasts. A** Cell percentage of each sample in the five fibroblast subtypes. KD1–KD6 were sampled from lesion regions, and KN1–KN6 were sampled from non-lesion regions. **B** Co-localization of α-SMA and CTHRC1 in non-lesion and lesion regions. The green color represents the expression of CTHRC1, while the red color represents the expression of α-SMA. **C** Flowchart depicts the sorting of CTHRC1 fibroblasts from tissues. **D** Trajectory analysis of the five fibroblast cell types. **E** Heatmap of top molecules in our trajectory analysis. The expressed genes were divided into cluster 1 and cluster 2 based on hcluster analysis. The variance in cluster1 was due to F02, while the variance in cluster 2 was due to F01.

**Fig. S3. The pathogenic role of CTHRC1+ fibroblasts in keloids. A** Cell infiltration in lesion regions based on R_O/E_ index. An index greater than 1 indicates that more cells were infiltrated in this region, while an index lower than 1 indicates fewer cells. **B** IHC staining of CTHRC1 in non-lesion and lesion regions of fibroblasts. The bar plot used to quantify the positive cells is shown below. The t-test was used in the plot. **C** The volcano plot indicates differential genetic programs in CTHRC1+ fibroblasts (F02) between non-lesion and lesion regions. **D** Bar plot of top significant molecules in C—including CTGF, COL1A1, COL1A2, CYR61, WISP2, and CTHRC1. **E** Pathway-enrichment analysis of differentially expressed genes in C. The dark color represents a higher fold-change. **F** Expression of pathways related to ECM deposition in non-lesion and lesion regions. Left panel depicts the EMT pathway, and the right the TGF-β pathway. **G** Trajectory analysis of all fibroblasts based on non-lesion and lesion regions. The red dots represent cells from the lesion region, and blue dots represent cells from the non-lesion region. **H** Top genes with pseudo-time (from the non-lesion region to the lesion region).

**Fig. S4. Regulons of CTHRC1+ fibroblasts. A** The mean expression of transcription factors in each fibroblast subtype. The color represents the relative level of transcription factors. **B** The distribution of RSS in each cell type. Different colors represent F01–F05 fibroblasts. **C** Top regulons with the highest RSS in CTHTC1+ fibroblasts. In the top-right plot, the blue color indicates the F02-CTHRC1 cells. In the bottom-right plot, the blue color depicts the range of transcriptional activities of CREB3L1. **D** The interaction network of 10 transcription modules from M1 to M10. The dark plot represents the CSI index as higher than 0.7. **E** The regulatory network of transcription factors from M9 (CREB3L1, CREB5, and JDP2) is based on the database. CREBBP/EP300 was found to be co-regulated by the three transcription factors. **F** Co-localization staining of COL1A1 and CREB3L1 in non-lesion and lesion regions. The green color represents the expression of COL1A1, while the red color represents the expression of CREB3L1. **G** IHC of CREB3L1 in non-lesion and lesion regions. Bar plot at right reflects the quantification of positive cells.

**Fig. S5. Identification of regulon modules in CTHRC1+ fibroblasts. A** Transcriptional activities of transcription factors in the five fibroblast subtypes. The red rectangle designates the CTHRC1+ fibroblasts. **B** RSS plots of F01, F03, F04, and F05. **C** Heatmaps of different transcription models based on their CSI indices. **D** The activities of 10 transcription models in the five fibroblast subtypes. **E** Binding motif of transcription factors from M9 and M10 modules. **F** Quantification of protein expression in the siCREB3L1 experiment. **G** Quantification of protein expression in the SGC-CBP30 experiment.

**Fig. S6. Roles of macrophages in the pathogenesis of keloids. A** Cellular infiltration of immune cells based on their R_O/E_ index. An index higher than 1 indicated that more cells infiltrated into the region, while an index less than 1 indicated fewer cells. **B** Giemsa, toluidine blue, and CPA3 staining of tissues from non-lesion and lesion regions, with IHC indicating mast cells. **C** Developmental stage of the three macrophage subtypes. **D** Pathway-enrichment analysis of the three macrophage subtypes between non-lesion and lesion regions. The red color represents up-regulated pathways, and the blue color represents down-regulated pathways. **E** Co-localization staining of CD68 and SPP1 is shown here, SPP1 was stained with the green color, while CD68 was stained with the red color. **F** Western blot of ECM proteins stimulated by SPP1 at different concentrations; the corresponding quantification plot is shown at the bottom.

1. Young MD, Behjati S. SoupX removes ambient RNA contamination from droplet-based single-cell RNA sequencing data. *Gigascience*. Dec 26 2020;9(12)doi:10.1093/gigascience/giaa151

2. Stuart T, Butler A, Hoffman P, et al. Comprehensive Integration of Single-Cell Data. *Cell*. Jun 13 2019;177(7):1888-1902 e21. doi:10.1016/j.cell.2019.05.031

3. McGinnis CS, Murrow LM, Gartner ZJ. DoubletFinder: Doublet Detection in Single-Cell RNA Sequencing Data Using Artificial Nearest Neighbors. *Cell Syst*. Apr 24 2019;8(4):329-337 e4. doi:10.1016/j.cels.2019.03.003

4. Korsunsky I, Millard N, Fan J, et al. Fast, sensitive and accurate integration of single-cell data with Harmony. *Nat Methods*. Dec 2019;16(12):1289-1296. doi:10.1038/s41592-019-0619-0

5. Zappia L, Oshlack A. Clustering trees: a visualization for evaluating clusterings at multiple resolutions. *Gigascience*. Jul 1 2018;7(7)doi:10.1093/gigascience/giy083

6. Qiu X, Hill A, Packer J, Lin D, Ma YA, Trapnell C. Single-cell mRNA quantification and differential analysis with Census. *Nat Methods*. Mar 2017;14(3):309-315. doi:10.1038/nmeth.4150

7. Farrell JA, Wang Y, Riesenfeld SJ, Shekhar K, Regev A, Schier AF. Single-cell reconstruction of developmental trajectories during zebrafish embryogenesis. *Science*. Jun 1 2018;360(6392)doi:10.1126/science.aar3131

8. Efremova M, Vento-Tormo M, Teichmann SA, Vento-Tormo R. CellPhoneDB: inferring cell-cell communication from combined expression of multi-subunit ligand-receptor complexes. *Nat Protoc*. Apr 2020;15(4):1484-1506. doi:10.1038/s41596-020-0292-x

9. Browaeys R, Saelens W, Saeys Y. NicheNet: modeling intercellular communication by linking ligands to target genes. *Nat Methods*. Feb 2020;17(2):159-162. doi:10.1038/s41592-019-0667-5

10. Aibar S, Gonzalez-Blas CB, Moerman T, et al. SCENIC: single-cell regulatory network inference and clustering. *Nat Methods*. Nov 2017;14(11):1083-1086. doi:10.1038/nmeth.4463

11. Suo S, Zhu Q, Saadatpour A, Fei L, Guo G, Yuan GC. Revealing the Critical Regulators of Cell Identity in the Mouse Cell Atlas. *Cell Rep*. Nov 6 2018;25(6):1436-1445 e3. doi:10.1016/j.celrep.2018.10.045

12. Cabili MN, Trapnell C, Goff L, et al. Integrative annotation of human large intergenic noncoding RNAs reveals global properties and specific subclasses. *Genes Dev*. Sep 15 2011;25(18):1915-27. doi:10.1101/gad.17446611

13. Fuxman Bass JI, Diallo A, Nelson J, Soto JM, Myers CL, Walhout AJ. Using networks to measure similarity between genes: association index selection. *Nat Methods*. Dec 2013;10(12):1169-76. doi:10.1038/nmeth.2728

14. Shannon P, Markiel A, Ozier O, et al. Cytoscape: a software environment for integrated models of biomolecular interaction networks. *Genome Res*. Nov 2003;13(11):2498-504. doi:10.1101/gr.1239303

15. Liu ZP, Wu C, Miao H, Wu H. RegNetwork: an integrated database of transcriptional and post-transcriptional regulatory networks in human and mouse. *Database (Oxford)*. 2015;2015doi:10.1093/database/bav095

16. Castro-Mondragon JA, Riudavets-Puig R, Rauluseviciute I, et al. JASPAR 2022: the 9th release of the open-access database of transcription factor binding profiles. *Nucleic Acids Res*. Jan 7 2022;50(D1):D165-D173. doi:10.1093/nar/gkab1113

17. Liu X, Chen W, Zeng Q, et al. Single-Cell RNA-Sequencing Reveals Lineage-Specific Regulatory Changes of Fibroblasts and Vascular Endothelial Cells in Keloids. *J Invest Dermatol*. Jan 2022;142(1):124-135 e11. doi:10.1016/j.jid.2021.06.010

18. Deng CC, Hu YF, Zhu DH, et al. Single-cell RNA-seq reveals fibroblast heterogeneity and increased mesenchymal fibroblasts in human fibrotic skin diseases. *Nat Commun*. Jun 17 2021;12(1):3709. doi:10.1038/s41467-021-24110-y

19. He H, Suryawanshi H, Morozov P, et al. Single-cell transcriptome analysis of human skin identifies novel fibroblast subpopulation and enrichment of immune subsets in atopic dermatitis. *J Allergy Clin Immunol*. Jun 2020;145(6):1615-1628. doi:10.1016/j.jaci.2020.01.042

20. Vorstandlechner V, Laggner M, Kalinina P, et al. Deciphering the functional heterogeneity of skin fibroblasts using single-cell RNA sequencing. *FASEB J*. Mar 2020;34(3):3677-3692. doi:10.1096/fj.201902001RR

21. Zou Z, Long X, Zhao Q, et al. A Single-Cell Transcriptomic Atlas of Human Skin Aging. *Dev Cell*. Feb 8 2021;56(3):383-397 e8. doi:10.1016/j.devcel.2020.11.002
